# A Novel Aquaporin 12-like Protein from *Chilo suppressalis*: Characterization and Functional Analysis

**DOI:** 10.3390/genes10040311

**Published:** 2019-04-21

**Authors:** Ming-Xing Lu, Jie Song, Jing Xu, Guirong Wang, Yang Liu, Yu-Zhou Du

**Affiliations:** 1 College of Horticulture and Plant Protection & Institute of Applied Entomology, Yangzhou University, Yangzhou 225009, China; lumx@yzu.edu.cn (M.-X.L.); 18061154097@163.com (J.S.); tczxyt@gmail.com (J.X.); 2 State Key Laboratory for Biology of Plant Diseases and Insect Pests, Institute of Plant Protection, Chinese Academy of Agricultural Sciences, Beijing 100000, China; wangguirong@caas.cn (G.W.); liuyangsdu@126.com (Y.L.); 3 Jiangsu Key Laboratory of Crop Genetics and Physiology/Co-Innovation Center for Modern Production Technology of Grain Crops, Yangzhou University, Yangzhou 225009, China

**Keywords:** aquaporin, *Chilo suppressalis*, function, physiology, temperature, humidity

## Abstract

Aquaporins (AQPs), which are members of the major intrinsic protein (MIP) family, play an important role in the transport of water and other small, uncharged solutes across membranes. In this study, we identified gene encoding two aquaporin 12-like (AQP12L) proteins, *CsAqp12L_v1* and *CsAqp12L_v2*, from *Chilo suppressalis*, a serious rice pest in Asia. Phylogenetic analysis indicated that *Cs*AQP12L_V1 and *Cs*AQP12L_V2 were grouped in a well-supported cluster that included other members of Lepidoptera. The two proteins are almost identical, except that *Cs*AQP12L_V1 lacks 34 amino acids that are present in *Cs*AQP12L_V2 at site 217. The qRT-PCR indicated that both *CsAqp12L* and *CsAqp12L_v2* were expressed in heads, epidermis, foregut, midgut, and hindguts, with the highest level of expression in hindguts, heads, and epidermis. Expression of *CsAqp12L* and *CsAqp12L_v2* was detected in all life stages and both sexes and was highest in first instar larvae and lowest in eggs. Expression of *CsAqp12L* and *CsAqp12L_v2* was not significantly altered by exposure to brief changes in temperature. There were no significant differences in the third instar larvae, male and female pupae, and female adults in response to adverse humidity. However, the mRNA level of *CsAqp12L* in the fifth instar larvae and *CsAqp12L_v2* in male adults was induced significantly by low humidity, respectively. Moreover, *Xenopus* oocytes injected with cRNAs of *Cs*AQP12L_V1 and *Cs*AQP12L_V2 showed no significant changes in permeability to water, glycerol, trehalose, or urea. The two *Cs*AQP12L variants likely localize to an intracellular location in *C. suppressalis* and may respond to novel stimuli.

## 1. Introduction

Membrane intrinsic protein (MIP), also termed aquaporins (AQPs), are of molecular mass 26–35 kDa and facilitate the passive movement of water and small molecules across cell membranes [1,2,3]. AQPs play an important role in the physiological functions in insects, for example, freeze and heat tolerance of insect could be elevated by AQPs, and the reproduction of insects could be regulated by AQPs [4,5,6,7,8]. AQPs exist in all kinds of organisms [9,10] and usually contain six transmembrane domains (TM) connected by five loops (A-E), with the N- and C-termini localized to the cytoplasm. AQPs contain two highly conserved NPA (Asn-Pro-Ala) motifs located at loops B and E that form short hydrophobic helices and insert halfway into the membrane from opposing sides, thus facing each other and participating in substrate selectivity [3,11]. Solute selectivity is determined by a constricted region on the extracellular side of the channel, which is formed by aromatic residues and an arginine (ar/R) selectivity filter [12,13]. AQPs in eukaryotic organisms can be segregated into four major groups, including the classic aquaporins, the aquaporin 8-type aquaamoniaporins, the unorthodox channels (aquaporin 11 and aquaporin 12L), and the aquaglyceroporins (Glps) [10,14]. The classical arthropod aquaporins function in the transportation of water, urea, and polyols, and include four major clades of insect aquaporins: The *Drosophila* integral protein (Drip), *Pyrocoelia rufa* integral protein (Prip), the big brain (Bib), and entomoglyceroporin (Eglp) [10]. Additional studies suggest that insects have aquaporins belonging to three major groups, which include the classical aquaporins, the Glps, and the unorthodox aquaporin 12-like (AQP12L) proteins [10,14]. However, compared to the other kinds of AQPs, the studies on the AQP12L are very few. Recently, several unorthodox AQP12L channels have been identified in insects [10,14,15,16,17]. However, the first authentic AQP12 was identified in human pancreatic acinar cells and did not localize to the plasma membrane when expressed in *Xenopus laevis* [18]. In insects, NPA motifs are replaced by other non-canonical amino acids, and the functions of AQP12L proteins are largely unknown.

The *Chilo suppressalis* (Walker) (Lepidoptera: Pyralidae) is an important pest of rice in Asia and causes huge damage in China, especially in the southern regions. *C. suppressalis* is confronted with adverse humidity present in rice fields throughout its development [19]; however, the first instar larvae and pupae are sensitive to drowning; irrigation in rice fields at the proper time is an important agricultural measure to control this pest. It is important to understand the underlying functions of aquaporins in the striped stem borer to demonstrate the mechanisms of water regulation. In this study, we characterized the genes encoding *C. suppressalis* AQP12L (*Cs*AQP12L), compared them with homologs in other insects, and assayed the abundance of *CsAqp12L* in different tissues, organs, and developmental stages. *CsAqp12L* mRNA expression levels in various developmental stages and sexes were investigated in response to different relative humidities and temperatures. To further explore the function(s) of *Cs*AQP12L, oocyte swelling assays were conducted using water and three kinds of solutes. Our results expand existing knowledge of AQPs in *C. suppressalis* and may ultimately provide insights for the control of *C. suppressalis* by the utilization of regulated mechanisms of their AQPs.

## 2. Materials and Methods

### 2.1. Insects

The population of *C. suppressalis* was collected from the suburb of Yangzhou (32.39° N, 119.42° E). Then, they were reared successively to the third generation on seedlings in a growth chamber at 27 ± 1 °C with a 16:8 h (L:D) photoperiod, and the relative humidity (RH) was 75 ± 5%.

### 2.2. RNA Isolation and Molecular Cloning of Full-Length CsAqp12L

According to the manufacturer’s process, total RNA of the fifth instar larvae of *C. suppressalis* was extracted using the SV Total RNA isolation system (Promega, Madison, WI, USA). Single-stranded cDNA was reverse-transcribed using an oligo (dT)_18_ primer (TaKaRa, Dalian, China). A partial sequence of the *CsAqp12L* gene was obtained from the ChiloDB database (http://www.insect-genome.com/data/detail.php?id=7) [20]. Touchdown PCR reaction conditions were as follows: 94 °C for 3 min, 19 cycles of 94 °C for 30 s, 65–45 °C (annealing temperature decreased by 1 °C/cycle, from 65 °C to a “touchdown” 45 °C) for 30 s, 72 °C for 1 min, and then 25 cycles of 94 °C for 30 s, 45 °C (annealing temperature) for 30 s, and 72 °C for 1 min, followed by extension at 72 °C for 10 min. Full-length cDNA of *CsAqp12L* was obtained by 5′- and 3′-RACE (SMART RACE, Clontech, USA), and then these full length cDNAs were confirmed by 5′-RACE (Table 1), in order to validate the accuracy of sequences of *CsAqp12*. All PCR products were purified using a gel extraction kit (Axygen, New York, NY, USA), and they were subcloned into pGEM-T Easy Vector (Promega, USA) and sequenced.

### 2.3. Sample Preparation and Exposure to Temperature and RH

To analyze expression patterns of *CsAqp12L* in various tissues and organs of *C. suppressalis*, fifth instar larvae of similar sizes were selected and dissected. Heads (HE), epidermis (EP), fat body (FB), foregut (FG), midgut (MG), hindgut (HG), Malpighian tubules (MT), and hemocytes (HC) of these larvae were dissected in 0.9% NaCl. Each sample included tissues and organs from 10 larvae of the fifth instar larvae and was replicated four times. To analyze expression in different developmental stages of *C. suppressalis*, the one-day-old eggs, first, second third, fourth, and fifth instar larvae, one-day-old pupae (male and female), and newly emerged adults (male and female) were collected separately; each stage included four replicates. All samples were frozen in liquid nitrogen and stored at −80 °C.

To explore the potential role of *CsAqp12L* in protecting *C. suppressalis* from temperature stress, expression patterns of *CsAqp12L* in fifth instar larvae were assessed under different temperatures for 2 h. The ten larvae were placed in glass tubes and exposed to selected temperatures (−9 °C, −8 °C, −6 °C, −3 °C, 0 °C, 27 °C, 30 °C, 33 °C, 36 °C, 39 °C, 42 °C, and 43 °C) for 2 h in a constant-temperature subzero incubator (Jiangnan Equipment, China). After the 2 h temperature treatment, larvae were allowed to recover for 2 h at 27 °C, then the surviving larvae were immediately recorded and frozen in liquid nitrogen and stored at −80 °C. Each treatment was repeated three times. At the same time, each treatment included at least four individual larvae.

We used fifth instar larvae, pupae (male and female), and adults (male and female) separately and exposed them to four different relative humidities (25%, 50%, 75%, and 95%) for 24 h; third instar larvae were exposed to the same RH concentrations for 12 h. RH levels were maintained using a controlled humidity chamber with ±1.5% RH accuracy (SANTN HTC-100, Shanghai, China) at 27 °C. The 30 individuals of *C. suppressalis* were treated with the same humidity, and surviving larvae were frozen in liquid nitrogen and stored at −80 °C. Each treatment included more than three individuals.

### 2.4. Real-Time Quantitative PCR (qPCR)

Total RNA from individuals of *C. suppressalis* was extracted by the SV Total RNA Isolation System (Promega, Madison, WI, USA) according to the manufacturer’s instructions. RNA purity was estimated by spectrophotometric measurements at 260 and 280 nm (Eppendorf, BioPhotometer plus, Germany). First-strand cDNA was reverse-transcribed with the Bio-Rad iScript cDNA Synthesis Kit. *CsAqp12L* primers (Table 1) for qPCR amplified *CsAqp12L_v1* and *CsAqp12L_v2* concurrently. *CsAqp12L_v2* primers specifically amplified *CsAqp12L_v2*. Real-time quantitative PCR was performed in a 20 μL reaction volume that contained iTaq Universal SYBR Green Supermix (10 μL; Bio-Rad, USA), cDNA templates (2 μL), gene specific primers (1 µL, see Table 1), and PCR-grade water (6 μL). All treatments included four biological replicates, and each reaction was run in triplicate. Reactions were carried out using a CFX-96 Real-Time PCR System (Bio-Rad Laboratories Inc., Hercules, CA, USA), and the specific conditions: 95 °C for 3 min, 40 cycles of 95 °C for 5 s, and 30 s at the Tm for the primer pair (Table 1). To determine the specificity of the amplification conditions and PCR products, a melting curve analysis from 65 to 95 °C was applied. Expression in different tissues and organs of larvae exposed to temperature stress were normalized using the genes encoding histone 3 (*H3*) and elongation factor 1 (*Ef1*) [21]. The expression of *CsAqp12L* in different developmental stages was normalized to *EF1*. The expression value of *CsAqp12L* in response to RH levels was normalized to *18S* (3rd instar larvae), *Actin* (fifth instar larvae), *Ubi* (female pupae and male adults), *Ef1* (female adults), and *Tub* (male pupae). Foregut tissue, one-day-old eggs, and larvae incubated at 27 ± 1 °C were used as controls (www.ambion.com/techlib/basics/rtpcr/index.html) [22]. The quantity of *CsAqp12L* mRNA was calculated using the 2^−ΔΔCt^ method.

### 2.5. Expression in Xenopus Oocytes

Vector construction for expression in *Xenopus* ooyctes has been described previously [23]. Using primers with a Kozak consensus sequence and *Spe*I and *Not*I restriction sites, the full-length coding regions of *CsAqp12L_v1* and *CsAqp12L_v2* were amplified with a high-fidelity polymerase (TaKaRa PrimeSTAR^®^ HS DNA polymerase, Kusatsu, Japan). The PCR products were digested with *Spe*I and *Not*I, subcloned into pT7Ts (Invitrogen, USA), and linearized with *Sma*I. The cRNAs encoding *Cs*AQP12L_V1 and *Cs*AQP12L_V2 were synthesized in vitro using the mMESSAGE mMACHINE T7 kit (Ambion, USA). Purified cRNAs were suspended in nuclease-free water at a concentration of 0.2 μg/μL and stored at −80 °C.

Stage V and VI oocytes were defolliculated with 2 mg/mL collagenase I (GIBCO, USA) in washing buffer (96 mM NaCl, 5 mM HEPES, 5 mM MgCl_2_, and2 mM KCl (pH 7.6)) for approximately 1 h at room temperature (26 °C). Oocytes were then cultured overnight at 18 °C and then microinjected with *CsAqp12L_v1*, *CsAqp12L_v2* separately, or nuclease-free water as a control (injection volume was 27.6 nL for cRNAs and water). After injection, oocytes were incubated for 3 days at 18 °C in 1X Ringer’s solution (96 mM NaCl, 5 mM MgCl_2_, 5 mM HEPES, 2 mM KCl, and 0.8 mM CaCl_2_ (pH = 7.6)) supplemented with 5% dialyzed horse serum, 50 mg/mL tetracycline, 100 mg/mL streptomycin, and 550 mg/mL sodium pyruvate.

Oocytes were transferred to a three-fold dilution of 1X Ringer’s solution in distilled water, and images were acquired of oocyte silhouettes every 15 s (up to 5 min) using a CCD camera (DP-72, Olympus Corp., Japan) of an Olympus SZX16 stereomicroscope. Osmotic water permeability (*P*_f_) was calculated as described previously [24,25] using the following equation: *P*_f_ = *V*_0_ × *d* (*V*/*V*_0_)/*dt*/(*S* × *V*_w_ × (Osm_in_–Osm_out_)), where *V*_0_ is the oocyte initial volume (*V*_0_ = 9 × 10^−4^ cm^3^), *S* is the oocyte surface area (*S* = 0.045 cm^2^), *V*_w_ is the molecular volume of water (*V*_w_ = 18 cm^3^/mol), Osm_in_ is 202 mmol·kg^−1^, and Osm_out_ is 59 mmol·kg^−1^. The relative oocyte volume (*V*/*V*_0_) was calculated from the relative area (*A*/*A*_0_) in the focal plane, *V*/*V*_0_ = (*A*/*A*_0_)^3/2^. Oocytes were also transferred to an isotonic solution containing 140 mM of various solutes (urea, trehalose, or glycerol) for solute transport assays. The volume changes were recorded for 5 min as described above. However, apparent solute permeability was calculated from the equation: *P*_sol_ = (*d* (*V*/*V*_0_)/*dt*) × (*V*_0_/*S*) [26,27]. Water and solute permeabilities were tested for at least nine different *Xenopus* oocytes.

### 2.6. Bioinformatic and Phylogenetic Analyses

Open reading frames (ORFs) were identified using ORF Finder (http://www.ncbi.nlm.nih.gov/gorf/gorf.html). The Compute pI/MW tool available at the ExPASy (http://web.expasy.org/compute_pi/) was used to predict molecular weight and pI. The analyses of transmembrane regions were executed in the TMHMM (http://www.cbs.dtu.dk/services/TMHMM-2.0/). The deduced amino acid sequences of *Cs*AQP12L proteins were aligned using ClustalX versus 1.83 (www.clustal.org/download/1.X/ftp-igbmc.ustrasbg.fr/pub/ClustalX/). Phylogenetic trees were constructed in the MEGA v. 7.0 [28] and MrBayes 3.2.2, respectively [29].

### 2.7. Data Analysis

The data were analyzed using SPSS 16.0 software (IBM, Armonk, NY, USA) and presented as means ± SE (standard error). A one-way analyses of variance (one-way ANOVA) followed by Tukey’s test were performed to compare differences between treatments.

## 3. Results

### 3.1. Sequence and Phylogenetic Analysis of CsAQP12L

**Figure 1:**
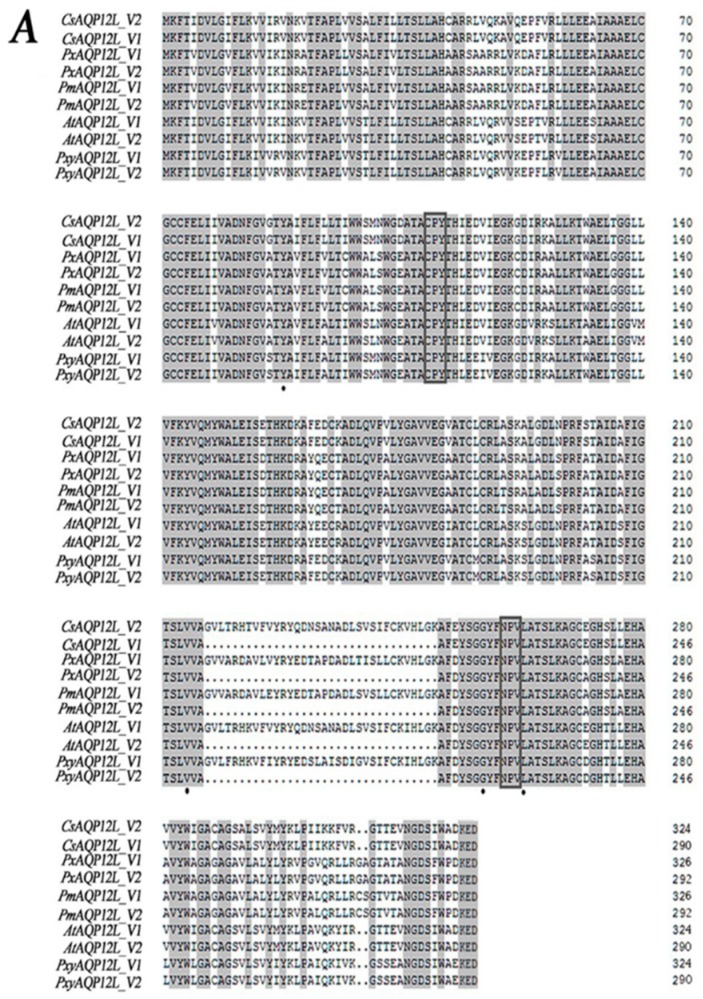

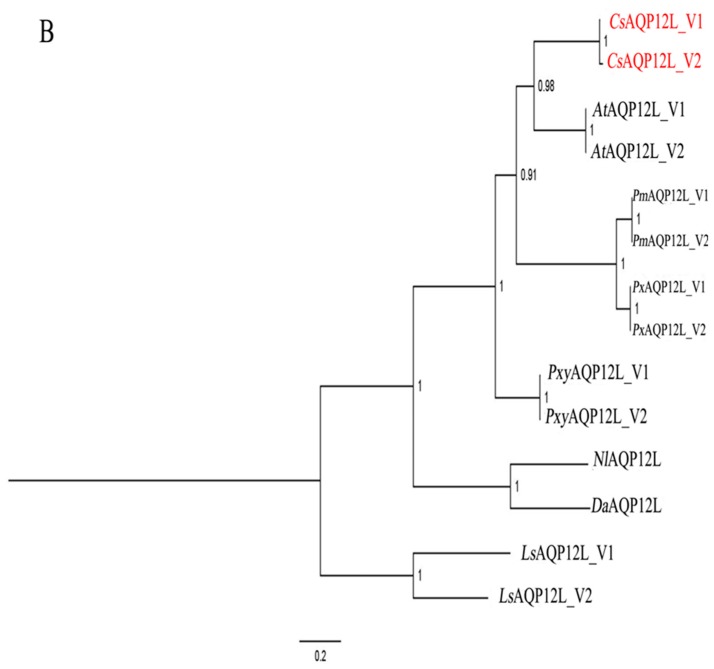
Comparative analysis of *Chilo suppressalis* AQP12L (*Cs*AQP12L) with AQP12L proteins in other insect species. (**A**) Multiple sequence alignment of *Cs*AQP12L_V1 and *Cs*AQP12L_V2 from *C. suppressalis* (*Cs*AQP12L_V1, MF033357; *Cs*AQP12L_V2, MF033358), *Papilio xuthus* (*Px*AQP12L_V1, XM_013311304; *Px*AQP12L_V2, XM_013311305), *Papilio machaon* (*Pm*AQP12L_V1, XM_014505979; *Pm*AQP12L_V2, XM_014505980), *Amyelois transitella* (*At*AQP12L_V1, XM_013345423; *At*AQP12L_V2, XM_013345424), *Plutella xylostella* (*Pxy*AQP12L_V1, XM_011560529; *Pxy*AQP12L_V2, XM_011560536). Conserved motifs are bordered with a rectangle and the ar/R selectivity site is indicated by black solid dots. (**B**) Phylogenetic analysis of AQP12L proteins using the Bayesian inference (BI) method. The number associated with each internal branch represents Bayesian posterior probabilities. The amino acid sequences used for construction of the phylogenetic tree were obtained from GenBank and were deposited under the following accession numbers: *Cs*AQP12L_V1 and *Cs*AQP12L_V2 (*C. suppressalis* see above), *Px*AQP12L_V1 and *Px*AQP12L_V2 (*P. xuthus*, see above), *Pm*AQP12L_V1 and *Pm*AQP12L_V2, (*P. machaon*, see above), *At*AQP12L_V1 and *At*AQP12L_V2 (*A. transitella*, see above), *Pxy*AQP12L_V1 and *Pxy*AQP12L_V2 (*P. xylostella*, see above), *Ls*AQP12L_V1 and *Ls*AQP12L_V2 (*Lepeophtheirus salmonis*, KR005665, KR005666), *H Nl*AQP12L (*Neodiprion lecontei*, XM_015664627), and *Da*AQP12L (*Diachasma alloeum*, XM_015259511).

We obtained two distinctly different cDNAs encoding *CsAqp12L*; *CsAqp12L_v1* was deposited in GenBank as accession no. MF033357 and *CsAqp12L_v2* as accession no. MF033358. *CsAqp12L_v1* consists of 1604 bp with a 873-bp coding sequence; the *Cs*AQP12L_V1 predicted protein is 31.84 kDa, contains 290 amino acids, and has a predicted pI of 5.6. *Cs*AQP12L_V2 is 1698 bp and has a 975-bp coding sequence; the predicted protein is 35.66 kDa, contains 324 amino acids, and has a pI of 6.18 (Figure S1 and S2). The two proteins are almost identical except that *Cs*AQP12L_V1 lacks 34 amino acids that are present in *Cs*AQP12L_V2 at site 217. This phenomenon was similar with that of other Lepidoptera. Both *Cs*AQP12L_V1 and *Cs*AQP12L_V2 have the non-conserved CPY (Cys-Pro-Tyr) and conserved NPV (Asn-Pro-Val) motifs associated with unorthodox members of the aquaporin superfamily, which is also true for *Bemisia tabaci* (Figure 1A) [12]. The ar/R constriction site in the two *Cs*AQP12L proteins differs from orthodox AQPs and is comprised of Tyr88, Val214, Gly (*Cs*AQP12L_V1, 223; *Cs*AQP12L_V2, 257), and Leu (*Cs*AQP12L_V1, 229; *Cs*AQP12L_V2, 263). Transmembrane (TM) topology varied for *Cs*AQP12L_V1 and *Cs*AQP12L_V2 according to the predictive method utilized; the proteins were projected to contain four (TMHMM), five (RHYTHM, HHMTOP), or seven (Pbobius, TOPCONS) membrane spanning segments. A comparison of deduced amino acid sequences showed that *Cs*AQP12L exhibited a high level of amino acid identity with AQP12L in other insects, including *Amyelois transitella* (89%)*, Plutella xylostella* (87%), *Papilio machaon* (82%), and *Papilio xuthus* (82%) (Figure 1A). The phylogenetic trees of *Cs*AQP12L and the other AQP12L have been constructed through the neighbor-joining, maximum likelihood, minimum evolution, maximum parsimony, and Bayesian inference methods, and five phylogenetic trees exhibited a consistent trend. The phylogenetic tree constructed indicated that *Cs*AQP12L was highly similar to AQP12L proteins in *A. transitella*; furthermore, Lepidopteran forms of AQP12L grouped together and were separated from orthologous proteins in Hymenoptera and Hemiptera. Two variants of AQP12L from every kind of organism fell into a well-supported cluster. (Figure 1B).

### 3.2. CsAqp12L Expression in Tissues and Organs of C. suppressalis

**Figure 2:**
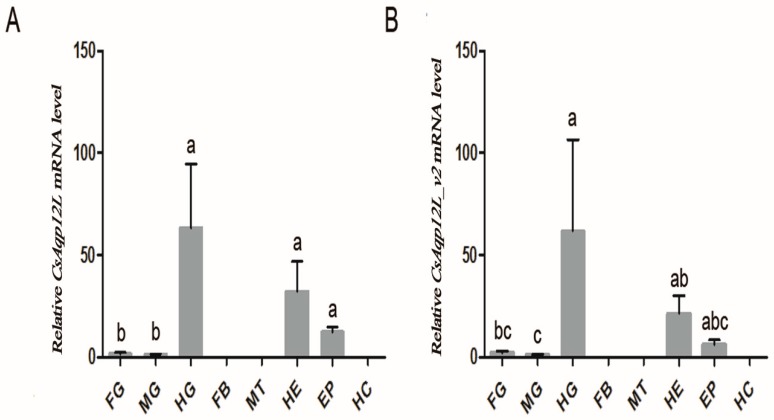
Expression of *CsAqp12L* in different tissues and organs of *Chilo suppressalis*. (**A**) *CsAqp12L*; (**B**) *CsAqp12L_v2*. Abbreviations: FG, foreguts; MG, midguts; HG, hindguts; FB, fat bodies; MT, Malpighian tubules; HE, head; EP, epidermis; and HC, hemocytes. Data represent mean ± SE for four replications. Means labeled with different letters are significantly different (*P* < 0.05, one-way ANOVA, Tukey).

Real-time quantitative PCR verified that *CsAqp12L* and *CsAqp12L_v2* mRNA were both expressed in the foregut, midgut, hindgut, heads, and epidermis of *C. suppressalis* larvae, but not in hemocytes, Malpighian tubules, or the fat body. Expression of *CsAqp12L_v* and *CsAqp12L_v2* was highest in the hindgut, followed by heads and epidermis (Figure 2A,B). The expression patterns of *CsAqp12L_v* and *CsAqp12L_v2* were similar (Figure 2B).

### 3.3. CsAqp12L Expression in Different Developmental Stages of C. Suppressalis

**Figure 3:**
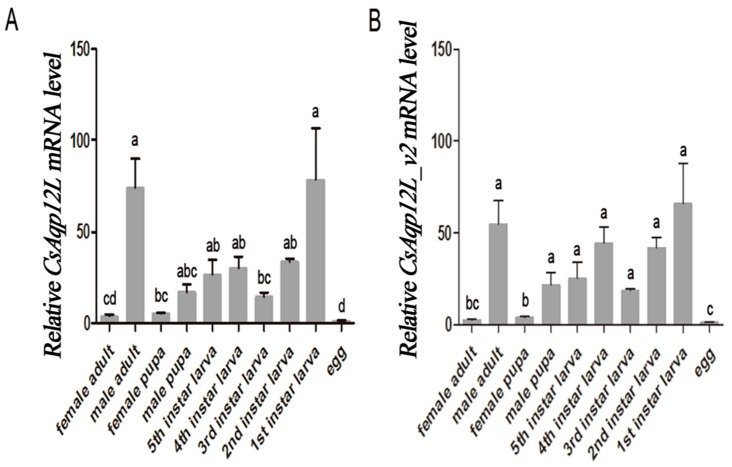
Expression of *CsAqp12L* genes in different developmental stages of *Chilo suppressalis*. (**A**) *CsAqp12L*; (**B**) *CsAqp12L_v2*. Data represent mean ± SE for four replications. Means labeled with different letters were significantly different (*P* < 0.05, one-way, Anova, Tukey).

Expression of *CsAqp12L* and *CsAqp12L_v2* was analyzed in different life stages and sexes of *C. suppressalis*. The qPCR verified that expression of both *CsAqp12L_v1* and *CsAqp12L_v2* was detected in all life stages and both sexes (Figure 3). Expression of *CsAqp12L_v* and *CsAqp12L_v2* was highest in the first instar larvae, followed by male adults, and was lowest in *C. suppressalis* eggs. Expression of *CsAqp12L_v* and *CsAqp12L_v2* was higher in male adults as compared to female adults, respectively (*F*_9__, 27_ = 15.937, *P* < 0.0001; *F*_9, 26_ = 24.775, *P* < 0.0001) (Figure 3).

### 3.4. CsAqp12L Expression in Response to Different RH Levels and Temperatures

**Figure 4:**
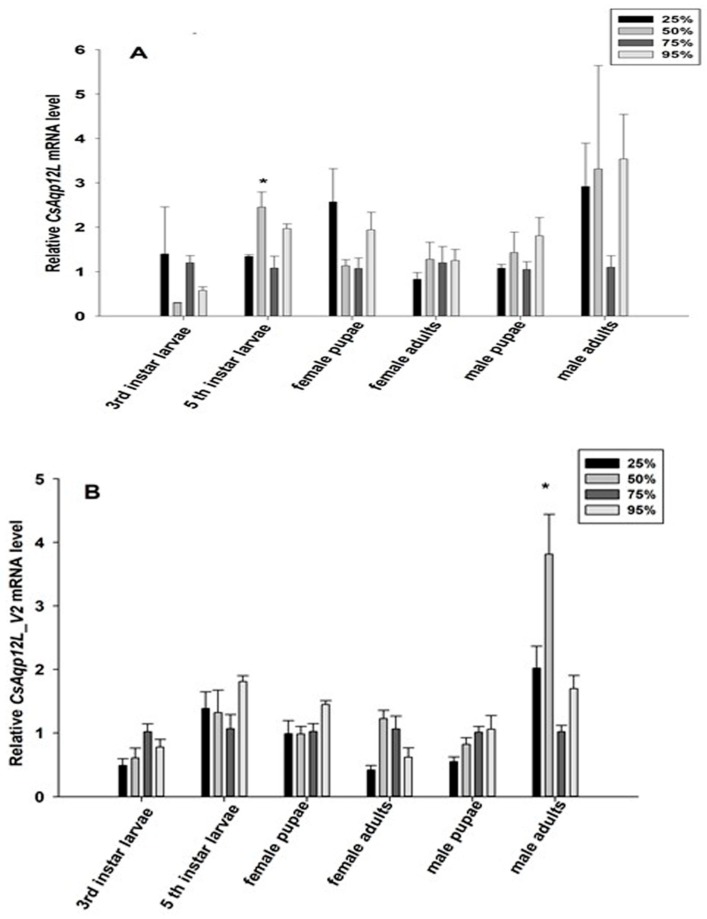
Expression of *CsAqp12L* genes from *Chilo suppressalis* in response to different levels of relative humidity. Third instar larvae were exposed to 25%, 50%, 75%, and 95% RH for 12 h. Fifth instar larvae, pupae, and adults were exposed to 25%, 50%, 75%, and 95% for 24 h. (**A**) Expression of *CsAqp12L* gene. (**B**) Expression of *CsAqp12L_v2* gene. Histograms indicate relative expression levels. Data represent mean ± SE for four replications. Means labeled with asterisks were significantly different (*P* < 0.05, one-way ANOVA, Tukey).

**Figure 5:**
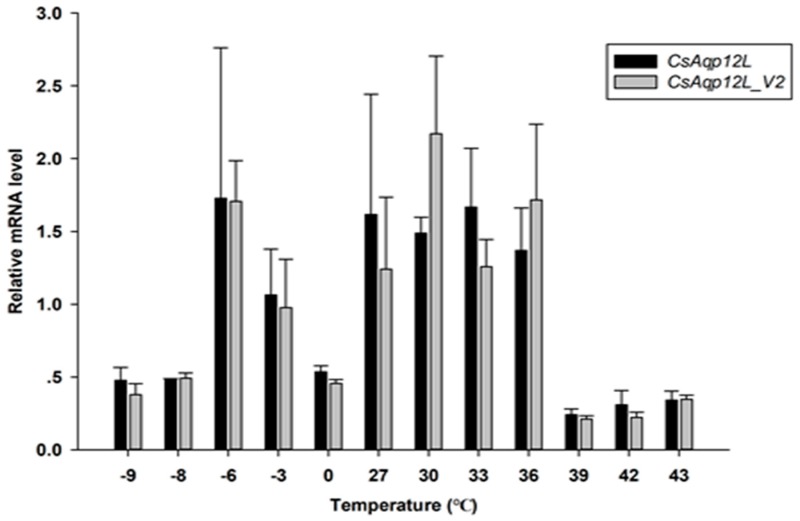
Expression of *CsAqp12L* and *CsAqp12L_v2* in larvae of *Chilo suppressalis* exposed to temperature stress. Larvae were exposed to −9 °C, −8 °C, −6 °C, −3 °C, 0 °C, 27 °C, 30 °C, 33 °C, 36 °C, 39 °C, 42 °C, and 43 °C for 2 h. Histograms indicate relative expression levels. Data represent mean ± SE for four replications. Means labeled with asterisks were significantly different (*P* < 0.05, one-way ANOVA, Tukey).

To determine whether RH impacted *CsAqp12L* and *CsAqp12L_v2* expression, fifth instar larvae, pupae, and adults were exposed to 25%, 50%, 75%, and 95% RH for 24 h; third instar larvae were exposed to the same RH conditions for 12 h. While the mRNA levels of *CsAqp12L* and *CsAqp12L_v2* were changed in response to different RH levels, these differences in the third instar larvae, male and female pupae, and female adults did not reach significance (*P* > 0.05). Moreover, the mRNA level of *CsAqp12L* in the fifth instar larvae was induced significantly by 50% RH (*F*_3__, 9_ = 7.036, *P* = 0.010), and the abundance of *CsAqp12L_v2* in male adults treated with 50% RH was higher than that of the other treatments of male adults (*F*_3__, 9_ = 12.210, *P* = 0.002) (Figure 4). The expression levels of *CsAqp12L* and *CsAqp12L_v2* after larvae were exposed to different temperatures were not induced significantly (*P* > 0.05) (Figure 5).

### 3.5. Water and Solute Permeability Assays in Xenopus Oocytes

**Figure 6:**
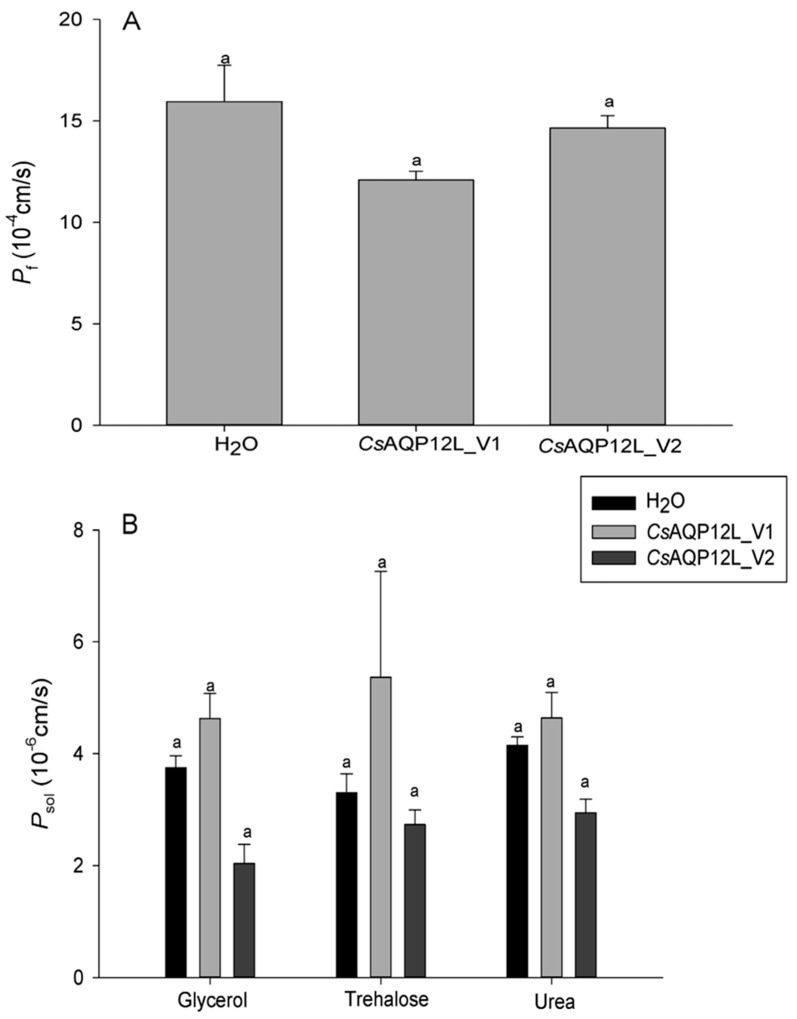
Functional assays of *Chilo suppressalis* aquaporin (*Cs*AQP12L). (**A**) Water permeability in *Xenopus* oocytes microinjected with *Cs*AQP12L_V1 cRNA, *Cs*AQP12L_V2 cRNA, and nuclease-free H_2_O (control). (**B**) Solute permeability assays in *Xenopus* oocytes exposed to 140 mM of glycerol, trehalose, and urea. Values represent the mean ± SE of oocytes.

The permeability of *Cs*AQP12L_V1 and *Cs*AQP12L_V2 with respect to water, glycerol, trehalose, and urea transport was performed using the *Xenopus* oocyte expression system. Our results showed that the osmotic permeability coefficients (*P*_f_) of *Cs*AQP12L_V1 and *Cs*AQP12L_V2 oocytes in water were 12.098e^−4^ cm/s and 15.377e^−4^ cm/s, respectively, and these values were not significantly different from the control (*F*_2, 25_ = 2.797, *P* > 0.05) (Figure 6A). No significant difference in glycerol, trehalose, or urea uptake was observed in *X. laevis* oocytes expressing *Cs*AQP12L_V1 and *Cs*AQP12L_V2 (Figure 6B); however, some oocytes showed a slight shrinkage in response to these solutes (*F*_2, 20_ = 2.749, *P* > 0.05; *F*_2, 23_ = 0.033, *P >* 0.05; *F*_2, 23_ = 2.700, *P* > 0.05) (Figure 6B).

## 4. Discussion

In this study, two transcriptional variants of genes encoding aquaporin 12-like proteins (*Cs*AQP12L_V1 and *Cs*AQP12L_V2) were identified and characterized from the striped stem borer, *C. suppressalis*. As with *Bemisia tabaci* and *Lepeophtheirus salmonis* [14,16], both *Cs*AQP12L_V1 and *Cs*AQP12L_V2 contain non-conserved CPY and NPV motifs. However, the second NPA motif is still NPA in *Lygus hesperus* [15]. Multiple sequence alignments of the deduced protein products indicated that *Cs*AQP12L_V1 and *Cs*AQP12L_V2 shared high identity with AQP12L from other insect species (Figure 1A). Phylogenetic analysis of AQP12L from other insect species revealed a distinct separation between the Lepidoptera and members of the Diptera, Hymenoptera, and Hemiptera (Figure 1B). Several lepidopteran insects have two transcriptional variants of AQP12L, and the *Cs*AQP12L_V2 variant encodes a protein with 34 additional amino acids. Interestingly, *Cs*AQP12L_V1 and *Cs*AQP12L_V2 could be due to splice variation from the same gene.

We found that *CsAqp12L* and *CsAqp12L_v2* were expressed in *C. suppressalis* foreguts, midguts, hindguts, heads, and epidermis, but not in the fat body, Malpighian tubules, or hemocytes (Figure 2). Our results indicated that *CsAqp12L* mRNA was abundantly expressed in the hindgut, which is also the case for *Aqp12L* in *Aedes aegypti* [30]. In *B. tabaci*, *BtAqp12L* transcripts were most abundant in immature white flies and adult guts [16]. The highest expressional level of the other AQP from *C. suppressalis* (*Cs*Drip1) was also found in the hindgut [31]. Therefore, we speculated the hindgut of *C. suppressalis* is an important organ for regulating the balance of water. Our result demonstrated the highest expression of *CsAqp12L* in first instar larvae, followed by male adults; expression was lowest in eggs, which was also the case for *Aqp12L* in *L. hespeus* [15]. The expression of *CsAqp12L_v* and *CsAqp12L_v2* was higher in male adults than that of female adults. We speculated that *CsAqp12L* expression might be more related to reproduction of male adults of *C. suppressalis*.

Several studies have indicated that AQP12L does not localize to the plasma membrane, but instead has an intracellular location and function [10,14,15,16,18,32]. In the present study, although no antibody was available to confirm that the protein was produced in the oocyte assays, oocytes expressing *Cs*Drip1 showed an eleven-fold increase in the osmotic water permeability coefficient (*P_f_*), compared to control oocytes in same expressional system [31]. However, no significant differences in permeability were observed in oocytes expressing *Cs*AQP12L_V1 or *Cs*AQP12L_V2 or oocytes injected with water (Figure 6A). At the same time, the mRNA levels of *CsAqp12L* and *CsAqp12L_v2* in the third instar larvae, male and female pupae, and female adults were not sensitive to relative humidity changes (Figure 4). In a previous study, expression of *PvAqp1* was dehydration-inducible and ubiquitous, whereas that of *PvAqp2* was dehydration-repressive and fat body-specific in *Polypedilum vanderplanki*. However, when expressed in *Xenopus* oocytes, *Pv*AQP1 and *Pv*AQP2 both transported water [33]. Thus, it can be seen different kinds of AQPs possessed various characteristics that take on different functions. The mRNA expression levels of *CsAqp12L* and *CsAqp12L_v2* also were not elevated significantly under different temperatures (Figure 5). We speculate that *Cs*AQP12L and *Cs*AQP12L_V2 have no obvious role in desiccation tolerance or temperature stress, possibly because of their presumed intracellular location. In mammals, AQP12 had an intracellular location in pancreatic cells and was speculated to function in digestive enzyme secretion [18]. The pancreas of AQP12 null mice showed enhanced response to a cholecystokinin analog (caerulein), and developed severe, acute pancreatitis [34]. A subsequent study suggested that AQP12 might function in the fusion of zymogen granules [35]. Thus, it is tempting to speculate that *Cs*AQP12L and *Cs*AQP12L_V2 in *C. suppressalis* might respond to novel, uncharacterized stimuli.

In our study, two *Cs*AQP12L variants were identified in *C. suppressali*. In *Anopheles gambiae*, aquaporin 1 was produced as two splice variants with distinctly different roles [36]. To date, some insect aquaporins have been identified as splice variants, and the rationale for this phenomenon is unclear. Future studies are underway to clarify the role of *Cs*AQP12L_V1 and *Cs*AQP12L_V2 in the physiology and development of *C. suppressalis*.

## Figures and Tables

**Table 1:** Primers used in this study.

Primer Name	Primer Sequences (5′ → 3′)	Tm (°C)	Length (bp)
**Cloning *Aqp12L* fragment**			
*Aqp12L-F*	GCCTTCATCGGGACTTCTTTA	65–45	325
*Aqp12L-R*	CGTTGACTTCTGTAGTGCCCC		
**5′- and 3′-Race PCR**			
*Aqp12L* 5′	TAGCAACCTGCCTATGTAGACTAGCCTC	68	941
*Aqp12L* 3′	AACTGGGGTGATGCGACAGCTTGTC		1225
**Vector construction**			
V-AQP12L-F	TCAACTAGTGCCACCATGAAGTTCACAATCGATGTATTG	68	873 + 975
V-AQP12L-R	TCAGCGGCCGCTTAATCTTCTTTGTCCGCCC		
**qRT-PCR**			
qPCR- *Aqp12L*-F	GGGGACTTGAATCCTCGGTT	57	125 + 227
qPCR- *Aqp12L*-R	CCTGCTTTCAGTGAGGTGGC		
qPCR- *Aqp12L_v2*-F	CGGTATCAGGACAATAGTGCCA	56.5	158
qPCR- *Aqp12L_v2*-R	GCGTGTTCCAACAGCGAGT		
*Ef1-F*	AAAATGGACTCGACTGAACCCC	56.6	137
*Ef1-R*	TCTCCGTGCCAACCAGAAATA		
*H3-F*	TGACGAAACCCCTTCGCTT	56	184
*H3-R*	CCCAGGTCGGTCTTGAAATCT		
*18S-F*	GTGATGGGACGAGTGCTTTTATT	62.5	258
*18S-R*	GCTGCCTTCCTTGGATGTGG		
*Actin-F*	AAAGAAACAGCAAAAGTCGGGG	56	243
*Actin-R*	GTTCAATGGAGGTTCGGTAAGTAAA		
*Ubi-F*	TCACCGACAGCAAACCAGACT	60.2	219
*Ubi-R*	GGAAGAAAACACCCCCCTCATATA		
*Tub-F*	GAGGGCATGGACGAGATGGA	60.4	178
*Tub-R*	ACGACGGTACGAGTATGACGGG		

## Supplementary Materials

The following are available online at https://www.mdpi.com/2073-4425/10/4/311/s1. Figure S1: The complete cDNA sequence and predicted amino acid sequence of *Cs*AQP12L_V1 and *Cs*AQP12L_V2 of *Chilo suppressalis*, Figure S2: The complete cDNA sequence and predicted amino acid sequence of CsAQP12L_V2 of *Chilo suppressalis*.
