# Supplementary material for: A Novel Aquaporin 12-like Protein from Chilo suppressalis: Characterization and Functional Analysis
Source: Genes (Basel). 2019 Apr 21;10(4):311. doi: 10.3390/genes10040311 (PMC6523266; doi:10.3390/genes10040311)
Supplement: Supplementary file 1 [file genes-10-00311-s001.pdf]

Figure S1. The complete cDNA sequence and predicted amino acid sequence of *Cs*AQP12L_V1 of *Chilo suppressalis*

```
1     ATGGGGGCACACCGATCGCCCGCACAATAAAAACCAAACGTAGTACCTATTATATTTTTCCTACGAATTCGACTTTCCCTGTGGTCACTT
91    GAGAAAGTGTCACGGGAAAAGGTACCTAAAGTGTTCTAAAATTCGGGTGTTGCAGTTCTAAAATGTACCTATGTGAAGTGATAAGGAAGT
181   GAATTTTGAAATTGGAAGGTAAATTAGACTGACGTCATTATGAAGTTCACAATCGATGTATTGGGGATTTTCTTGAAAGTTGTCATTAGG
1                                                    M  K  F  T  I  D  V  L  G  I  F  L  K  V  V  I  R
271   GTAAACAAAGTTACGTTTGCACCATTAGTGGTATCAGCGCTGTTCATACTCCTCACGTCTCTCCTGGCCCACTGCGCGCGGCGCCTGGTG
18     V  N  K  V  T  F  A  P  L  V  V  S  A  L  F  I  L  L  T  S  L  L  A  H  C  A  R  R  L  V
361   CAGAAGGCAGTGCAGGAGCCTTTCGTCAGATTACTGTTGGAGGAGGCGATCGCAGCTGCCGAACTATGCGGATGTTGCTTCGAACTCATT
48     Q  K  A  V  Q  E  P  F  V  R  L  L  L  E  E  A  I  A  A  A  E  L  C  G  C  C  F  E  L  I
451   ATTGTGGCTGACAACTTCGGGGTGGGAACGTACGCCATATTCCTGTTCTTGCTGACAATCTGGTGGTCCATGAACTGGGGTGATGCGACA
78     I  V  A  D  N  F  G  V  G  T  Y  A  I  F  L  F  L  L  T  I  W  W  S  M  N  W  G  D  A  T
541   GCTTGTCCGTACACTCATATCGAGGATGTGATTGAAGGCAAAGGTGATGTTCGAAAAGCTTTGCTAAAAACTTGGGCGGAATTGACTGGC
108    A  C  P  Y  T  H  I  E  D  V  I  E  G  K  G  D  V  R  K  A  L  L  K  T  W  A  E  L  T  G
631   GGTCTTCTGGTATTTAAATACGTCCAAATGTATTGGGCTCTTGAAATATCTGAGACGCATAAAGATAAGGCTTTTGAAGACTGCAAAGCC
138    G  L  L  V  F  K  Y  V  Q  M  Y  W  A  L  E  I  S  E  T  H  K  D  K  A  F  E  D  C  K  A
721   GATCTACAGGTACCAGTATTATATGGAGCAGTCGTAGAAGGTGTAGCAACCTGCCTATGTAGACTAGCCTCCAAAGCTTTAGGGGACTTG
168    D  L  Q  V  P  V  L  Y  G  A  V  V  E  G  V  A  T  C  L  C  R  L  A  S  K  A  L  G  D  L
811   AATCCTCGGTTCTCAACCGCTATCGATGCCTTCATCGGGACTTCTTTAGTCGTCGCCGCTTTTGAATATTCCGGTGGATATTTCAACCCG
198    N  P  R  F  S  T  A  I  D  A  F  I  G  T  S  L  V  V  A  A  F  E  Y  S  G  G  Y  F  N  P
901   GTTCTGGCCACCTCACTGAAAGCAGGATGCGAAGGTCACTCGCTGTTGGAACACGCAGTTGTCTATTGGATCGGTGCTTGCGCTGGATCT
228    V  L  A  T  S  L  K  A  G  C  E  G  H  S  L  L  E  H  A  V  V  Y  W  I  G  A  C  A  G  S
991   GCATTATCAGTTTATATGTACAAACTACCCATCATCAAGAAGTTTGTCCGTGGCACTACAGAAGTCAACGGTGATAGTATTTGGGCGGAC
258    A  L  S  V  Y  M  Y  K  L  P  I  I  K  K  F  V  R  G  T  T  E  V  N  G  D  S  I  W  A  D
1081  AAAGAAGATTAAAATGTATCATTTCCTGTTTTTGTACATTTTCCAAGTCATATTCCAGTTTCTTTTCTTGTCTTCTTCTGTTGTTCATTA
288    K  E  D  *
1171  TTTAAGTTAACTTCATTATCTCTCTTTATAAACTAAGAAGACTTAAGAAGAACGTTATCTGATAATTAATTTATTAAAGTGATACCTTAC
1261  TCTAGTCAAGTTATATCTCTAAGTTAAACCTCGTGCTTTGTCCATTATACAGATTTTACAAGTTATTGTATTAAAGGAAACAAAAAATAC
1351  GCAAACTTTTATATGAAATTTTTTTACTTATATTTTTTTATAATTTATTGTAAATTCGCCTTGATGTCATCACACAATTGAATATTGACG
1441  TTTTGAACTGTGAATAATTGATTTCGTTTAAGGTTTGATTGTATAGCGTCATTTGTTACTATGCTTCCATTGAAATCTAACCTAAATGTG
1531  TTCCAAAAATATAATAAGTGCTACGTGAACCTGTTGTAAATAAATCCAATGTTTTGTTAAAAAAAAAAAAAAA
```

Figure S2. The complete cDNA sequence and predicted amino acid sequence of *Cs*AQP12L_V2 of *Chilo suppressalis*

```
1     AAGCAGTGGTATCAACGCAGGTACATGGGGGTAGTACCTATTATATTTTTCCTACGAATTCGACTTTCCCTGTGGTCACTTGAGAAAGTG
91    TCACGGGAAAAGGTACCTAAAGTGTTCTAAAATTCGGGTGTTGCAGTTCTAAAATGTACCTATGTGAAGTGATAAGGAAGTGAATTTTGA
181   AATTGGAAGGTAAATTAGACTGACGTCATTATGAAGTTCACAATCGATGTATTGGGGATTTTCTTGAAAGTTGTCATTAGGGTAAACAAA
1                                                  M  K  F  T  I  D  V  L  G  I  F  L  K  V  V  I  R  V  N  K
271   GTTACGTTTGCACCATTAGTGGTATCAGCGCTGTTCATACTCCTCACGTCTCTCCTGGCCCACTGCGCGCGGCGCCTGGTGCAGAAGGCA
21     V  T  F  A  P  L  V  V  S  A  L  F  I  L  L  T  S  L  L  A  H  C  A  R  R  L  V  Q  K  A
361   GTGCAGGAGCCTTTCGTCAGATTACTGTTGGAGGAGGCGATCGCAGCTGCCGAACTATGCGGATGTTGCTTCGAACTCATTATTGTGGCT
51     V  Q  E  P  F  V  R  L  L  L  E  E  A  I  A  A  A  E  L  C  G  C  C  F  E  L  I  I  V  A
451   GACAACTTCGGGGTGGGAACGTACGCCATATTCCTGTTCTTGCTGACAATCTGGTGGTCCATGAACTGGGGTGATGCGACAGCTTGTCCG
81     D  N  F  G  V  G  T  Y  A  I  F  L  F  L  L  T  I  W  W  S  M  N  W  G  D  A  T  A  C  P
541   TACACTCATATCGAGGATGTGATTGAAGGCAAAGGTGATGTTCGAAAAGCTTTGCTAAAAACTTGGGCGGAATTGACTGGCGGTCTTCTG
111    Y  T  H  I  E  D  V  I  E  G  K  G  D  V  R  K  A  L  L  K  T  W  A  E  L  T  G  G  L  L
631   GTATTTAAATACGTCCAAATGTATTGGGCTCTTGAAATATCTGAGACGCATAAAGATAAGGCTTTTGAAGACTGCAAAGCCGATCTACAG
141    V  F  K  Y  V  Q  M  Y  W  A  L  E  I  S  E  T  H  K  D  K  A  F  E  D  C  K  A  D  L  Q
721   GTACCAGTATTATATGGAGCAGTCGTAGAAGGTGTAGCAACCTGCCTATGTAGACTAGCCTCCAAAGCTTTAGGGGACTTGAATCCTCGG
171    V  P  V  L  Y  G  A  V  V  E  G  V  A  T  C  L  C  R  L  A  S  K  A  L  G  D  L  N  P  R
811   TTCTCAACCGCTATCGATGCCTTCATCGGGACTTCTTTAGTCGTCGCCGGAGTCCTCACTAGGCACACGGTTTTCGTCTACCGGTATCAG
201    F  S  T  A  I  D  A  F  I  G  T  S  L  V  V  A  G  V  L  T  R  H  T  V  F  V  Y  R  Y  Q
901   GACAATAGTGCCAATGCGGACTTAAGCGTGTCCATCTTTTGCAAAGTACATTTAGGAAAAGCTTTTGAATATTCCGGTGGATATTTCAAC
231    D  N  S  A  N  A  D  L  S  V  S  I  F  C  K  V  H  L  G  K  A  F  E  Y  S  G  G  Y  F  N
991   CCGGTTCTGGCCACCTCACTGAAAGCAGGATGCGAAGGTCACTCGCTGTTGGAACACGCAGTTGTCTATTGGATCGGTGCTTGCGCTGGA
261    P  V  L  A  T  S  L  K  A  G  C  E  G  H  S  L  L  E  H  A  V  V  Y  W  I  G  A  C  A  G
1081  TCTGCATTATCAGTTTATATGTACAAACTACCCATCATCAAGAAGTTTGTCCGTGGCACTACAGAAGTCAACGGTGATAGTATTTGGGCG
291    S  A  L  S  V  Y  M  Y  K  L  P  I  I  K  K  F  V  R  G  T  T  E  V  N  G  D  S  I  W  A
1171  GACAAAGAAGATTAAAATGTATCATTTCCTGTTTTTGTACATTTTCCAAGTCATATTCCAGTTTCTTTTCTTGTCTTCTTCTGTTGTTC
321    D  K  E  D  *
1261  ATTATTTAAGTTAACTTCATTATCTCTCTTTATAAACTAAGAAGACTTAAGAAGAACGTTATCTGATAATTAATTTATTAAAGTGATACC
1351  TTACTCTAGTCAAGTTATATCTCTAAGTTAAACCTCGTGCTTTGTCCATTATACAGATTTTACAAGTTATTGTATTAAAGGAAACAAAAA
1441  ATACGCAAACTTTTATATGAAATTTTTTTACTTATATTTTTTTATAATTTATTGTAAATTCGCCTTGATATCATCACACAATTGAATATT
1531  GATGTTTTGAACTGTGAATAATTGATTTCGTTTAAGGTTTGATTGTATAGCGTCATTTGTTACTATGCTTCCATTGAAATCTAACCTAAA
1621  TGTGTTCCAAAAATATAATAAGTGCTACGTGAACCTGTTGTAAATAAATCCAATGTTTTGTTAGTCAAAAAAAAAAAA
```
